# Recent Advances in Environmentally Friendly and Green Degumming Processes of Silk for Textile and Non-Textile Applications

**DOI:** 10.3390/polym14040659

**Published:** 2022-02-09

**Authors:** Lei Zhu, Junxiong Lin, Liujun Pei, Yuni Luo, Dali Li, Zhichao Huang

**Affiliations:** 1 Engineering Research Centre for Eco-Dyeing and Finishing of Textiles, Ministry of Education, Zhejiang Sci-Tech University, Hangzhou 310018, China; linjunxiong@zstu.edu.cn; 2 Key Laboratory of Advanced Textile Materials and Manufacturing Technology, Ministry of Education, Zhejiang Sci-Tech University, Hangzhou 310018, China; 3 College of Textile Science and Engineering (International Institute of Silk), Zhejiang Sci-Tech University, Hangzhou 310018, China; 4 Engineering Research Center of Textile Chemistry and Clean Production, Shanghai University of Engineering Science, Shanghai 201620, China; peilj@sues.edu.cn; 5 School of Textiles and Fashion, Shanghai University of Engineering Science, Shanghai 201620, China; 6 Key Lab of Science & Technology of Eco-Textile, Ministry of Education, College of Chemistry, Chemical Engineering and Biotechnology, Donghua University, Shanghai 201620, China; yuniluo@163.com; 7 Department of Bioengineering, Nanjing University of Science and Technology, Nanjing 210094, China; lidali@njust.edu.cn

**Keywords:** silk filaments, fibroin, sericin, green degumming, enzyme, evaluation, properties

## Abstract

Silk has been widely used not only in the textile field but also in non-textile applications, which is composed of inner fibrous protein, named fibroin, and outer global protein, named sericin. Due to big differences, such as appearance, solubility, amino acid composition and amount of reactive groups, silk fibroin and sericin usually need to be separated before further process. The residual sericin may influence the molecular weight, structure, morphology and properties of silk fibroin, so that degumming of silk is important and necessary, not only in textile field but also in non-textile applications. Traditional textile degumming processes, including soap, alkali or both, could bring such problems as environmental damage, heavy use of water and energy, and damage to silk fibroin. Therefore, this review aims to present a systematic work on environmentally friendly and green degumming processes of raw silk, including art of green degumming process, quantitative and qualitative evaluation, influence of degumming on molecular weight, structure, morphology and properties of silk. It is anticipated that rational selection and design of environmentally friendly and green degumming process is quite important and meaningful, not only for textile application but also for non-textile application.

## 1. Introduction

Silk, generally known as the “queen of fiber”, has not only been used in the textile field but also in biomedical field [1,2,3]. In fact, silk can be produced by the species from Arachnida or Lepidoptera, such as mites, butterflies and moths [4]. Among such kinds of silks, those from the domesticated silkworms (*Bombyx* *mori*) are used mostly. Unless otherwise stated, silk in this paper points to the *Bombyx* *mori* silk.

The silk is mainly composed of two kinds of proteins, the inner insoluble fibrous protein, which is usually named fibroin, and the outer global hydrophilic protein, named sericin [2,5]. Due to big differences, such as appearance, solubility, amino acid composition and amount of reactive groups, silk fibroin and sericin usually needs to be separated before further processing.

In textile fields, in order to obtain silk fibroin filaments with an excellent hand, elegant luster, high capillary rise height, a process called degumming (also called scouring) is necessary [6,7]. Sometimes, when the whiteness of silk fibroin filaments is not good enough, bleaching is applied [6]. After degumming, silk fibroin filaments can be dyed, printed or finished for textile applications. While in non-textile field, purified silk fibroin can be obtained through a simple degumming process, such as alkali degumming and boiling water. After that, silk fibroin can be further processed to film, sponge, scaffold, hydrogel, and non-woven mats for non-textile applications.

Up to now, there are a few reviews reported on the degumming of silk materials. However, few reviews present the systematic work on environmentally friendly and green degumming process of silk for both textile and non-textile applications. Here, a systematic work focuses on the art of green degumming process, quantitative and qualitative evaluation, influence of degumming on molecular weight, structure, morphology and properties of silk.

## 2. Art of Degumming

Traditional degumming processes could cause problems such as environmental damage, heavy use of water and energy, and damage to silk fibroin. Therefore, this review presents some environmentally friendly and green degumming processes, such as enzyme degumming, CO_2_ supercritical fluid (CSCF) degumming, acid degumming, steaming degumming and ultrasonic degumming. Among such degumming processes, enzyme degumming is mostly used.

### 2.1. Enzyme Degumming

Enzymes, usually composed of amino acids, are one kind of large biological molecules biocatalyst, which can be widely used in textile field, such as pretreatment of cotton, degumming of silk, bleaching and shrink proofing of wool due to their mild conditions of temperature and pH, high specificity and efficiency, reduced water and energy consumption [8,9] and little damage to silk fibroin [10,11,12,13]. It is reported that more than three quarters of industrial enzymes are hydrolytic in action, while protein-degrading enzymes count for two over five [8]. Among protein-degrading enzymes, proteases are the largest group with animal, plant and microbial sources which could be active under alkaline, acidic and neutral conditions [9]. 

In order to completely separate sericin from silk fibroin without obvious hydrolytic damage to the latter, Li et al. [11] used papain to degum *Bombyx* *mori* silk filaments under nearly neutral conditions. They found that sericin was completely removed, and silk fibroin still had a high molecular weight when the concentration of papain reached 3.0 g/L. This could be explained because, here, papain specifically breaks the binding sites between L-arginine or L-lysine residue and another amino acid residue in sericin, resulting in the clean and smooth surface of silk fibroin. Furthermore, they also found higher tensile strength using papain degumming than that under the traditional degumming process with sodium carbonate. They further prove that papain is a good alternative for degumming silk.

Besides, Promboon et al. [14] selected a commercial grade stem bromelain as the effective degumming agent for Mai 1 silk filaments. The results indicated that the fibroin was not damaged, and the silk fabric was provided with good physical properties, such as tensile strength with bromelain degumming method, compared with traditional sodium carbonate degumming method. Although bromelain is shown as a good biocatalyst, active pH range and corresponding mechanism of action were not reported.

Freddi et al. [15] picked up four such commercial proteases for silk degumming process including oxidative-stable endopeptidase, bacterial high alkaline strain, papain and aspergillus pepsin I. They found that only alkaline and neutral proteases were effective for the degumming of silk filaments, while the acid protease was ineffective under the experimental conditions adopted. Further study showed that even though the degumming ratio reached 25%, there was almost no sericin remaining on the warp of silk filaments while still some sericin deposited on the highly twisted weft, indicating that texture of silk could also influence the effect of degumming.

Recently, Chim-anage et al. [16] screened and isolated an extracellular serine protease of *Bacillus* sp. C4 SS-2013 (C4), used for the degumming of silk filaments. They found that the protease has a high specificity to sericin protein, and even at incubation for three days, the silk fibroin was not damaged while the sericin was completely removed. Furthermore, this protease was easily concentrated and suitable for longer storage at low temperatures. Although C4 is a promising alternative for degumming silk, whether it can be in large-scale production and for wide applications is still unknown.

Apart from the above teams devoted to studying the biological degumming of silk filaments with enzymes, other groups also promote this art [17,18,19,20,21,22,23,24,25,26,27]. It is anticipated that with the discovery, screen and isolation of more enzymes suitable for the silk degumming, corresponding environmentally friendly and green degumming processes will be developed and make silk degumming flourish.

### 2.2. CSCF Degumming

CO_2_ supercritical fluid (CSCF) can be considered as the CO_2_ above its critical temperature (Tc, generally 304.25 K) and critical pressure (Pc, generally 7.38 MPa), under which CO_2_ shows some unique properties, such as appropriate viscosity and diffusivity like gas, appropriate density and solvating properties like liquid, making it as solvent candidate so that CSCF can be applied in many fields [28,29,30,31,32,33,34,35,36,37]. In textile fields, CSCF is usually used for dyeing due to its environmentally friendly nature for the replacement of organic solvents or water and easy recovery and recycling [28,29,30], compared with traditional dyeing process. Besides the application in dyeing synthetic or natural fibers, CSCF can also be used for pretreatment of cotton [34,35] and flax fibers [36,37]. However, up to now, little information on degumming of silk using CSCF was reported.

Lo and his collaborator [32,33] conducted a series of studies on silk degumming using CSCF. The whole process includes the acid pretreatment of silk filaments with the aid of a surfactant, treatment with CSCF in the container under appropriate conditions and post-treatment using ultrasonic method. In this way, the cleaned silk fibroin filaments could be obtained. The mechanism could be explained that after pretreatment with citric acid or tartaric acid, the silk filaments carry positive charges due to both the isoelectric point (pI) of silk fibroin (3.6) and sericin (3.3) higher than experiment pH (2–3) [31,32,33]. Since citric acid or tartaric acid contains a carboxylic acid group, they can interact with the hydrogen ion (proton) on the surface of silk sericin to damage the amino acid structure of the sericin and with the help of a surfactant, a hydrophilic site will be created under CSCF. Then sericin can be easily removed from silk filaments by ultrasonic post-treatment. Although silk degumming using CSCF is efficient and can keep silk fibroin less damaged than conventional ammonium hydroxide degumming method, the complicated process is required. Hence, the development of easy and efficient degumming process of silk filaments with less damage to silk fibroin by CSCF method is quite necessary.

### 2.3. Acid Degumming

Besides enzymes and CSCF degumming, degumming of silk filaments with citric acid can also be considered an environmentally friendly degumming process due to the biodegradable nature, reduced water consumption and less damage to silk fibroin [38,39]. Citric acid (CA) is a mild organic acid with good biodegradability, safe and pleasant taste, high water solubility, good chelating and buffering properties which has been widely used in food, cosmetic, chemical and biomaterials fields [38,39,40,41].

In biomaterials field, citric acid acts as a green cross-linker for various applications, such as tissue engineering, cancer therapy, wound dressings [42,43]. It is interesting to point out that, in textile field, citric acid was first used as the cross-linker for the textile finishing instead of pretreatment [44,45,46,47].

Tsukada et al. [48] applied different concentration of citric acid for the degumming of silk filaments to study the effect of citric acid treatment on structure, morphology and properties of silk filaments. They found that molecular conformation and the crystalline structure did not change after degumming with citric acid, and almost no sericin remained on the surface of silk filaments with 30% citric acid when the total weight loss reached 25.4%, together with good tensile properties. They stated that citric acid degumming can be an alternative for industrial application. However, the pH of degumming bath containing 30% citric acid was not reported and whether such conditions can be suitable for industrial applications is still unknown.

### 2.4. Steaming Degumming

As one kind of efficient processing methods for the biomass conversion, the used steam has higher efficiency of heat transfer due to its greater heat capacity and not decreasing the moisture content of treated objects like wood, compared with hot air [49,50,51]. In the textile wet process, steam treatment is often used for padding dyeing, printing and finishing process [52,53,54,55,56]. 

Similar to CSCF degumming, the steam process for textiles seems to be the environmentally friendly due to no harmful chemicals use and low water consumption. However, litter information on steaming process for pretreatment of silk filaments. Recently, Zhu et al. [57] showed a routine of silk degumming by steam treatment without aid of any chemicals. They used a modified pressure cooker as the steam treatment apparatus. Ultrasonic treatment and following washing process was applied after the steam degumming. The results show that sericin was almost completely removed under optimal conditions, and some physicochemical properties of the silk fibroin filaments did not change. Energy efficiency analysis indicates steam treatment is an efficient technique for raw silk degumming with lower processing cost and without chemical used, compared to the conventional chemical degumming methods. Since steam degumming for raw silk filaments can be considered as an environmentally friendly and green process, large scale of steam degumming of silk filaments for textile applications and non-textile applications is worthy of further investigation.

### 2.5. Ultrasonic Degumming

In biomedical applications, sonication becomes a useful tool to control the rapid sol-gel transition of silk fibroin to form hydrogel, and to regulate the protein structure to obtain protein-base materials [58,59,60]. In textile field, ultrasonication is also widely used for dye extraction, textile dyeing due to the ability of sonication of breaking aggregates of dyes, breaking the fiber-dye interfacial layer and increasing the swelling of fiber to accelerate their diffusion into the fiber [61,62,63].

Besides, sonication is also often seen in textile washing, including pretreatment and post-treatment [57,64,65,66]. With this technique, fewer chemicals are used and washing effectiveness is improved [64,65,66], therefore, ultrasonication can be thought to be an environmentally friendly and green process.

However, up to now, there is less information on degumming of silk filaments with sonication [67,68]. Recently, Arami et al. [67] applied different degumming techniques based on ultrasonication for raw silk yarns. In short, such techniques can be divided into two groups: one-bath degumming process and two-bath process, and the former group includes ultrasound degumming, ultrasound–enzyme degumming and traditional soap-alkali degumming, while the latter includes ultrasound and soap degumming, ultrasound and enzyme degumming, ultrasound and enzymes mixture degumming. The results show that the optimal degumming process is two-bath based ultrasound and enzymes mixture degumming, with significantly increased degumming efficiency perhaps due to their synergetic effect. Such a sonication-based environmentally friendly degumming process is very meaningful and important because it can help other research teams try different combinations of degumming process, develop many eco-friendly degumming processes and achieve a wide range of applications. Similar to the work by Arami, Li et al. [68] applied the citric acid, sodium carbonate and papain as the degumming agents for the silk filaments reeled from silk cocoons with the aid of ultrasonic treatment at four different frequencies. They found that a higher degumming rate was obtained degummed by ultrasonication at a lower frequency than at a higher frequency. They also found that papain degumming was more effective than citric acid and sodium carbonate with higher degumming rate. With increasing degumming temperature and time, less sericin was remained on the surface of silk filaments with papain degumming, resulting in smooth and clean surface, however, this may decrease silk whiteness. Sonication-based environmentally friendly degumming process is very meaningful and important because it can help other research teams try different combinations of degumming process, develop many eco-friendly degumming processes and achieve a wide range of applications.

In order to make cost and technical comparison with conventional degumming process, advantages and limitations of art of environment-friendly silk degumming are listed in Table 1. Although conventional soap, alkali or soap-alkali degumming displays their advantages of simple process and wide application, chemicals cannot be recycled, silk fibroin may be damaged, and demand of water and energy is high. For comparison, art of enzyme, CO_2_ supercritical fluid, acid, steam and ultrasonic degumming shows different advantages and limitations.

Besides the previously mentioned green degumming processes, ozone degumming [69,70,71,72], microwave irradiation degumming [31,73], green nonionic surfactant degumming [74] can also be considered as green degumming process. On the other hand, traditional alkali degumming with Na_2_CO_3_ is not considered as green degumming process, however, Bucciarelli et al. [75] optimized the alkali degumming process with Na_2_CO_3_ by design of experiment (DOE) and successfully removed all sericin from the silk fibroin with less salt, water, and energy, compared with traditional alkali degumming method, stating that it is possible to make this technique overall more environmentally sustainable. Therefore, traditional degumming process may be developed to become environmentally friendly technique by well-designed experiments.

## 3. Evaluation of Degumming

In order to determine whether the sericin has been completely removed from silk filaments, quantitative and qualitative evaluation will be used. Degumming ratio (sometimes called weight loss/WL), whiteness index, and capillary rise height are usually used as quantitative evaluation while Picric Acid-Carmine Staining (PACS) method as qualitative evaluation.

### 3.1. Quantitative Evaluation

#### 3.1.1. Degumming Ratio (DGR)

In the works relative to the degumming of silk no matter for textile applications or for non-textile applications, DGR or WL is introduced to give the evaluation of degumming effect. Actually, DGR and WL have the same meaning.

Generally, silk filaments can adsorb water from the air due to the hydrophilic groups on sericin and silk fibroin. When the sericin is not removed from silk filaments, many more water molecules can be adsorbed due to more hydrophilic groups on sericin. Therefore, it should be pointed out that the weight of silk materials in the dry state before and after degumming process is adopted in order to reduce calculation errors.

Since DGR is easy to obtain, it is usually adopted, together with other evaluations as a base for the judgment of complete removal of sericin. 

#### 3.1.2. Whiteness Index (WI)

Before degumming, due to appearance of the sericin, wax, dyes and impurities, the silk filaments have the lower WI. Once the sericin, wax, dyes and some impurities are almost completely removed, silk filaments have higher WI [17,20,22,68,76,77,78], and possesses attractive surface luster and smooth feel [79,80,81]. Therefore, in textile field, WI can also be used for evaluation of degumming effect.

In our previous work [82], coarse denier silk fabrics were degummed, and WI measured by whiteness meter WB-80 was used for evaluation of degumming effect. The results showed that with the increase in treatment of temperature (from 100 °C to 115 °C), the WI increased correspondingly, however, when the temperature was higher than 115 °C, WI decreased, which could be due to the slight yellowness resulting from the higher temperature.

Recently, Li et al. [68] adopted Hunter Whiteness Index (HWI) for evaluation of degumming effect. HWI can be determined using a Datacolor 650 and calculated by an equation [83]. They found that after degumming at 60 °C, HWI s obviously increased. At a temperature of 60 °C, the highest fiber whiteness values and clearer and brighter fiber surface result from papain degumming at 60 min, which could be due to complete removal of sericin from raw silk filaments [68].

#### 3.1.3. Capillary Rise Height (CRH)

Long et al. [7] applied a capillary rise measurement apparatus to measure the capillary rise height of raw silk fabric for evaluation of degumming effect. According to the difference in warp and weft directions, a silk fabric strip was cut in both directions and the capillary rise height was recorded after 30 min based on the literature [38]. The results showed that higher capillary rise height was obtained when the process argon gas pressure was kept at 80 Pa compared to that of traditional method.

Recently, Yu et al. [20] also carried out the measurement of capillary rise height of raw silk fabric to evaluate degumming effect according to the literature [84]. They found that with the introduction of natural tea saponin, the DGR was almost unchanged while the capillary rise height of silk fabric significantly increased. They stated that natural tea saponin as the natural surfactant, could effectively remove grease and wax on the surface of silk fabric so that the capillary rise height greatly increased.

### 3.2. Qualitative Evaluation

#### Picric Acid-Carmine Staining (PACS) Method

Picric acid and carmine staining (PACS) method [11,82,85,86,87] is based on the blend solutions of picric acid and carmine under alkali conditions and the principle can be shown that silk fibroin only adsorbs picric acid molecules in alkaline solution, showing a yellow color while sericin adsorbs both picric acid and carmine molecules simultaneously, displaying a red color. Generally, the red color can cover the yellow color resulting in the final red color. If sericin still remains the outer surface of silk fibroin, the overall color of the silk is red. The deeper the red color, the more sericin residues. When sericin has been completely removed, the silk shows the yellow color. Therefore, PACS is an effective qualitative evaluation of the degumming effect. This method is often used together with the result of DGR to evaluate whether the sericin has been removed completely.

**Figure 1:**
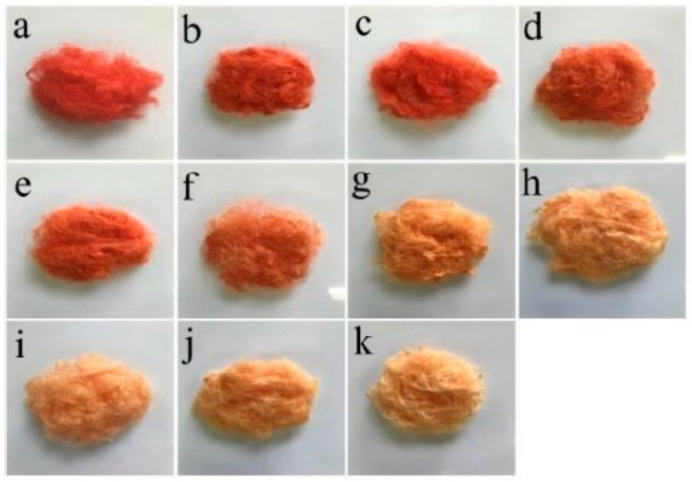
Color change of silk samples after picric acid and carmine staining: (**a**) Non-degummed raw silk, (**b–j**) degummed with 0.01, 0.05, 0.1, 0.3, 0.5, 1.0, 3.0, 4.0, and 6.0 g/L papain, respectively, (**k**) degummed with 0.5 g/L Na_2_CO_3_. Ref. [11].

Li et al. [11] used different concentrations of papain to degum silk filaments under nearly neutral conditions using traditional 0.5 g/L Na_2_CO_3_ as a control (Figure 1). The results showed that the undegummed silk filaments displayed the dark red (Figure 1a) while the traditional Na_2_CO_3_ degummed showed the yellow color (Figure 1k), indicating that sericin had been removed. Increasing concentration of papain would result in the red color slowly disappeared and the yellow color gradually appeared, indicating the residual sericin slowly decreased (Figure 1b–j).

## 4. Influence of Degumming

Since sericin is partially or completely removed by different degumming art, molecular weight, structure, morphology and properties of silk filaments would make some changes. Therefore, characterization of molecular weight, structure, morphology and properties of silk filaments may reflect the influence of degumming process.

### 4.1. Influence of Degumming on Molecular Weight of Silk Filaments

Generally, Sodium Dodecyl Sulfate-Polyacrylamide Gel Electrophoresis (SDS-PAGE) is effective for the measurement of molecular weight of soluble silk fibroin [88,89].

Víllora et al. [88] evaluate the effect of the degumming process on the molecular weight (MW) of the silk fibroin by SDS-PAGE. They found that compared with native silk fibroin with respective molecular weights are 391 kDa (heavy chain), 26 kDa (light chain), and 24 kDa (glycoprotein, P25), silk fibroin degummed by autoclave showed a light smear along the whole lane, and clear bands at 26 and 15 kDa can be observed, while silk fibroin degummed by long alkaline boiling (120 min) presented a size distribution as a smear between 100 and 10 kDa with a darker zone in the 30–40 kDa, and no bands were detected at 26 or 24 kDa, indicating that autoclave degumming gave the least aggressive treatment for silk fibroin while long alkaline boiling did the most aggressive treatment [88].

In general, the hydrogel properties of polymers are partially determined by MW [89]. Therefore, Kaplan et al. [89] systematically studied the effect of silk degumming time (DT) on the properties of enzymatically crosslinked silk hydrogels, in which they carried out PAGE measurement. For brevity, they classify the silk fibroin into two groups: one is high molecular weight from 1 min treatment in boiling Na_2_CO_3_ solution, the other is low molecular weight from 30- or 120-min treatment in the same solution. The results showed that various degumming time could bring different molecular weight of silk fibroin, for example, degumming time of 1, 30 and 120 min with corresponding molecular weight of 391 kDa (high), 157 kDa (medium) and 79 kDa (low), respectively, verify that degumming time controls the molecular weight of silk fibroin, i.e., longer degumming time results in lower molecular weight and vice versa.

### 4.2. Influence of Degumming on Structure of Silk Filaments

In order to investigate the effect of degumming process on the secondary structure (conformation) and crystalline structure of silk filament, a few groups applied Fourier transform infrared spectra (*FTIR*) and X-ray diffraction (*XRD*) for it [22,48,57,68,90].

Um et al. [90] carried out the degumming process of silk cocoons and studied the influence of degumming on structure of silk filament by *FTIR* and *XRD*. They found that when DGR was about 26%, indicating almost all the sericin removed, the degummed silks exhibited IR absorption peaks at 1620 (amide I band) and 1515 cm^−1^ (amide II band), together with a shoulder peak at 1260 cm^−1^ (amide III band) attributed to the β-sheet conformation. Furthermore, *XRD* measurements of all these degummed silks showed three diffraction peaks at 9.3°, 20.0°, and 23.9°, attributed to the β-sheet crystallite. They stated that since sericin is an amorphous material while silk fibroin contains highly crystallized regions, when almost all the sericin was removed, the β-sheet structure would increase correspondingly.

Li et al. [68] took silk cocoon as a starting material, investigating the influence of degumming on the structure of silks by *FTIR* and *XRD*. *FTIR* showed that the silks degummed with papain at 90 °C for 60 min using ultrasonication exhibited absorption peaks at 1640 cm^−1^ (amide I) attributed to random coil conformation, 1515 cm^−1^ (amide II), and 1227 cm^−1^ (amide III) attributed to β-sheet conformation. *XRD* showed that these silks exhibit peaks at 9.2° and 20.5°, attributed to β-sheet structure and at 28.6°, attributed to α-helical structures. They stated that the conformation and crystalline structure of silks did not change a lot by papain degumming under ultrasonication.

### 4.3. Influence of Degumming on Morphology of Silk Filaments

For investigating the effect of the degumming process on morphology of silk filament, some groups carried out SEM observation [11,48,68,87].

Li et al. [11] investigate the effect of degumming process by SEM observation. The undegummed silk fibroin was surrounded by a layer of sericin. The outer sericin decreased with the increase in papain concentration, especially when the concentration exceeded 4.0, the silk fibroin could be slightly damaged by an overly degumming process. On the other hand, under traditional Na_2_CO_3_ degumming process, the silk fibroin could suffer severe damage. Therefore, SEM observation could help to evaluate the degumming effect. Tsukada et al. [48] investigate the surface morphology of degummed silks by SEM observation. For the undegummed silk filament, SEM micrographs showed sericin appeared as a partially non-uniform coating on the surface of silk filament. When the silk filament was degummed by citric acid or soap solutions, the surface of silk filament is highly smooth, showing perfect degumming and that the silk filament is not damaged.

### 4.4. Influence of Degumming on Properties of Silk Filament

#### 4.4.1. Influence of Degumming on Mechanical Properties of Silk Filament

In order to evaluate the effect of silk degumming with alkyl polyglycoside (APG) on mechanical properties, Zhang et al. [74] carried out tensile tests of single filament. The results showed that degummed by traditional Na_2_CO_3_ method, the single filament had a maximum load of 4.902 cN and shift of 2.097 mm, while by traditional neutral soap method, the single filament had a maximum load of 5.619 cN and shift of 2.133 mm. For comparison, degummed by APG method, the maximum load and shift of silk filament were 5.400 cN and 2.276 mm, respectively. These results indicated that the traditional Na_2_CO_3_ method brought serious damage to the silk filament while APG degumming neutral soap degumming did not do so.

Promboon et al. [14] also carried out measurement of tensile strengths of the silk filament degummed with different concentrations of bromelain and compared it with traditional Na_2_CO_3_ method. The results showed that when the concentration of bromelain was 4, 5 and 6 g/L, tensile strengths were 5.49 ± 0.43, 4.76 ± 0.48 and 7.84 ± 0.38 N, respectively, significantly higher than that with traditional Na_2_CO_3_ method (4.02 ± 0.72 N), while the elongations at break of the silk samples with bromelain method were slightly lower than with traditional Na_2_CO_3_ method.

#### 4.4.2. Influence of Degumming on Thermal Properties of Silk Filaments

Víllora et al. [88] evaluated the effect of different degumming processes on the thermal properties of the silk fibroin by thermal gravimetric analyzer (TGA). The weight residue percentage of all the silk samples decreased quickly after 315–319 °C due to the degradation of silk fibroin, resulting from thermal degradation of β-Sheet ordered structures. Compared with silk cocoons with decomposition rate temperature at 330 °C, silk filaments degummed by autoclave and short alkaline boiling (30 min) showed middle Tdm at 319 °C, while silk filaments degummed by long alkaline boiling (120 min) and ultrasonication showed the lowest decomposition rate temperature at 315 and 316 °C, respectively.

Tsukada et al. [48] investigated thermal properties of silk samples with different degumming methods by differential scanning calorimetry (DSC). They found that a single predominant endothermic transition of DSC curve of undegummed silk filament appeared at 316 °C, starting at around 270 °C, attributed to the thermal decomposition of the silk fibroin with β-sheet conformation, and did not change with different degumming methods.

#### 4.4.3. Influence of Degumming on Dyeing Behavior of Silk Filaments

In the traditional textile industry, the degummed silk filaments are usually used for further wet process, such as dyeing, printing and finishing. Therefore, the effect of degumming process on wet process of silk filament is an interesting and meaningful topic [17,18,22,24,25,32,33,48].

Anis et al. [18] first obtained silk filaments with three degumming methods, i.e., enzyme method, high temperature (HT) method and traditional soap-alkali method. Then they applied an exhaustion dyeing method with acid and reactive dye. The results showed that the color strength (K/S value) of silk samples with the three degumming methods was similar and K/S value increased correspondingly when the dye concentration increased. The washing fastness of silk samples with the three degumming methods was also similar and decreased slightly with the increase in dye concentration. Although Anis et al. systematically investigated the effect of degumming process on the dying performance of silk filament, dye-fixation rate of reactive dye at different concentrations with the three degumming methods was not reported.

Tsukada et al. [48] studied dye behavior of silk filaments by different degumming methods with two kinds of acid dyes. The results showed that the dyed silks had comparatively lower dye uptake value, compared with raw silk filaments. They stated that sericin was an amorphous material with many amino groups on the surface of the raw silk filaments, resulting in faster and more dye sorption than the fully degummed silk.

## 5. Conclusions

A systematic work on environmentally friendly and green degumming processes of raw silk, including art of green degumming process, quantitative and qualitative evaluation, influence of degumming on molecular weight, structure, morphology and properties of silk is reviewed. The art of enzyme, CO_2_ supercritical fluid, acid, steaming and ultrasonic degumming are presented in detail. In fact, some previous non-environmentally friendly degumming processes can be improved, and new environmentally friendly and green degumming techniques can be developed. In future work, other new environmentally friendly degumming processes may be introduced in detail. It is anticipated that rational selection and design of environmentally friendly and green degumming process is quite important and meaningful, not only for textile application but also for non-textile application. Industrial applications of environmentally friendly and green degumming techniques are still the big challenge, while reduced production and environmental cost will be a crucial part for industrial applications in the future.

## Figures and Tables

**Table 1:** Comparison of art of environment-friendly silk degumming with the conventional.

Art	Advantages	Limitations	References
Enzyme	Mild conditions	Relatively high cost	[11,12,13,14,15,16,17,18,19,20,21,22,23,24,25,26,27]
	More choice of enzymes	Easy deactivation	
	Little damage to fibroin High specificity and efficiency		
CO_2_ supercritical fluid	Recycling of CO_2_	High requirements for equipment	[32,33]
	Little damage to fibroin	Demand of acid pretreatment and ultrasonic post-treatment	
Acid	Smooth and clean surface	Slightly decreased dye uptake percentage	[48,68]
	Increased tensile strength		
Steam	Relative lower cost	Lack of industrial application	[57]
	No addition of chemicals		
Ultrasonic	Improved degumming efficiency	Demand of addition of soap, alkali, acid or enzyme	[38,67,68]
	Reduced use of water and chemical	Low conversion of electrical to acoustical energy	
Conventional soap, alkali or soap-alkali	Simple process Wide application	Unrecyclable chemicals	[38,57,67,68]
		Damage to fibroin High demand of water and energy	

## Data Availability

Data sharing is not applicable to this article as no new data were created or analyzed in this study.

## References

[1] Konwarh R. (2019). Can the venerated silk be the next-generation nanobiomaterial for biomedical-device designing, regenerative medicine and drug delivery? Prospects and hitches. Bio-Des. Manuf..

[2] Vepari C., Kaplan D.L. (2007). Silk as a biomaterial. Prog. Polym. Sci..

[3] Kundu B., Kurland N.E., Bano S., Patra C., Engel F.B., Yadavalli V.K., Kundu S.C. (2014). Silk proteins for biomedical applications: Bioengineering perspectives. Prog. Polym. Sci..

[4] Kaplan D.L., Mello S.M., Arcidiacono S., Fossey S., Senecal K.W.M. (1998). Protein Based Materials.

[5] Zhou C.Z., Confalonieri F., Medina N., Zivanovic Y., Esnault C., Yang T., Jacquet M., Janin J., Duguet M., Perasso R. (2000). Fine organization of Bombyx mori fibroin heavy chain gene. Nucleic Acids Res..

[6] Wang J., Sun K. (1984). Principle of Dyeing and Finishing.

[7] Long J.J., Wang H.W., Lu T.Q., Tang R.C., Zhu Y.W. (2008). Application of Low-Pressure Plasma Pretreatment in Silk Fabric Degumming Process. Plasma Chem. Plasma Process..

[8] Choudhury A.R. (2014). Sustainable Textile Wet Processing: Applications of Enzymes, Roadmap to Sustainable Textiles and Clothing.

[9] Naveed M., Nadeem F., Mehmood T., Bilal M., Anwar Z., Amjad F. (2021). Protease—A Versatile and Ecofriendly Biocatalyst with Multi-Industrial Applications: An Updated Review. Catal. Lett..

[10] Thakur N., Goyal M., Sharma S., Kumar D. (2018). Proteases: Industrial applications and approaches used in strain improvement. Biol. Forum—Int. J..

[11] Feng Y., Lin J., Niu L., Wang Y., Cheng Z., Sun X., Li M. (2020). High Molecular Weight Silk Fibroin Prepared by Papain Degumming. Polymers.

[12] Araujo R., Casal M., Cavaco-Paulo A. (2008). Application of enzymes for textile fibres processing. Biocatal. Biotransform..

[13] Kim J., Kwon M., Kim S. (2016). Biological Degumming of Silk Fabrics with Proteolytic Enzymes. J. Nat. Fibers.

[14] Ninpetch U., Tsukada M., Promboon A. (2015). Mechanical Properties of Silk Fabric Degummed with Bromelain. J. Eng. Fibers Fabr..

[15] Freddi G., Mossotti R., Innocenti R. (2003). Degumming of silk fabric with several proteases. J. Biotechnol..

[16] Suwannaphan S., Fufeungsombut E., Promboon A., Chim-Anage P. (2017). A serine protease from newly isolated *Bacillus* sp. for efficient silk degumming, sericin degrading and colour bleaching activities. Int. Biodeterior. Biodegrad..

[17] Toprak T., Anis P., Akgun M. (2020). Effects of environmentally friendly degumming methods on some surface properties, physical performances and dyeing behaviour of silk fabrics. Ind. Textila.

[18] Anis P., Toprak T., Yener E., Capar G. (2019). Investigation of the effects of environmentally friendly degumming methods on silk dyeing performance. Text. Res. J..

[19] Chen J.H., Chen X., Zhang X.Y., Lan G.Q. (2016). Process technology for using papain protease in fresh cocoon degumming and reeling. Sci. Sericult..

[20] Wu C., Wang J., Li X., Yu Z. (2017). Research on scouring process of silk fabric with papain Q. Adv. Text. Technol..

[21] Gulrajani M., Agarwal R., Chand S. (2000). Degumming of silk with a fungal protease. Indian J. Fibre Text..

[22] Vyas S.K., Shukla S.R. (2015). Comparative study of degumming of silk varieties by different techniques. J. Text. Inst..

[23] Krishnaveni V. (2010). Study on effect of proteolytic enzyme degumming on dyeing of silk. Colourage.

[24] Nakpathom M., Somboon B., Narumol N. (2009). Papain enzymatic degumming of Thai Bombyx mori silk fibers. J. Microsc. Soc. Thail..

[25] Ibrahim N., El Hossamy M., Nessim A., Hassan T. (2007). Performance of bio-degumming versus conventional degumming processes. Colourage.

[26] Gowda K., Padaki N.V., Sudhakar R., Subramani R. (2007). Eco-friendly preparatory process for silk: Degumming by protease enzyme. Man-Made Text. India.

[27] Gulrajani M., Gupta S.V., Gupta A., Suri M. (1996). Degumming of silk with different protease enzymes. Indian J. Fibre Text..

[28] Banchero M. (2020). Recent advances in supercritical fluid dyeing. Color. Technol..

[29] AbouElmaaty T., Abd El-Aziz E. (2018). Supercritical carbon dioxide as a green media in textile dyeing: A review. Text. Res. J..

[30] Knez Z., Markocic E., Leitgeb M., Primozic M., Hrncic M.K., Skerget M. (2014). Industrial applications of supercritical fluids: A review. Energy.

[31] Rastogi S., Kandasubramanian B. (2020). Processing trends of silk fibers: Silk degumming, regeneration and physical functionalization. J. Text. Inst..

[32] Lo C.H. (2021). Degumming silk by CO_2_ supercritical fluid and their dyeing ability with plant indigo. Int. J. Cloth. Sci. Technol..

[33] Lo C.H., Chao Y. (2017). Degumming of silk fibers by CO_2_ supercritical fluid. J. Mater. Sci. Chem. Eng..

[34] Liu S.Q., Chen Z.Y., Sun J.P., Long J.J. (2016). Ecofriendly pretreatment of grey cotton fabric with enzymes in supercritical carbon dioxide fluid. J. Clean. Prod..

[35] Shi W., Liu S.Q., Sun J.P., Long J.J. (2018). A strategy for environmentally-friendly removal of impurities from cotton based on biocatalytic reaction in supercritical carbon dioxide. Cellulose.

[36] Zhang J., Zheng H.D., Zheng L.J. (2018). Effect of treatment temperature on structures and properties of flax rove in supercritical carbon dioxide. Text. Res. J..

[37] Zhang J., Zheng H.D., Zheng L.J. (2017). A Novel Eco-Friendly Scouring and Bleaching Technique of Flax Rove Using Supercritical Carbon Dioxide Fluid. J. Eng. Fibers Fabr..

[38] DeBari M.K., King C.I., Altgold T.A., Abbott R.D. (2021). Silk Fibroin as a Green Material. ACS Biomater. Sci. Eng..

[39] Sharma A., Kumar A., Kapoor A., Kumar R., Gangal S.V., Gangal V., Makhijani S.D. (1996). Assessment of biodegradability of organic acids by a defined microbial mixture. Bull. Environ. Contam. Toxicol..

[40] Mores S., de Souza Vandenberghe L.P., Júnior A.I.M., de Carvalho J.C., de Mello A.F.M., Pandey A., Soccol C.R. (2020). Citric acid bioproduction and downstream processing: Status, opportunities, and challenges. Bioresour. Technol..

[41] Berovic M., Legisa M. (2007). Citric acid production. Biotechnol. Annu. Rev..

[42] Salihu R., AbdRazak S.I., Zawawi N.A., Kadir M.R.A., Ismail N.I., Jusoh N., Mohamad M.R., Nayan N.H.M. (2021). Citric acid: A green cross-linker of biomaterials for biomedical applications. Eur. Polym. J..

[43] Wang M., Guo Y., Xue Y., Niu W., Chen M., Ma P.X., Lei B. (2019). Engineering multifunctional bioactive citric acid-based nanovectors for intrinsical targeted tumor imaging and specific siRNA gene delivery in vitro/in vivo. Biomaterials.

[44] Yang C.Q. (1993). Effect of pH on nonformaldehyde durable press finishing of cotton fabric: FT-IR spectroscopy study: Part I: Ester crosslinking. Text. Res. J..

[45] Yang C.Q. (1993). Effect of pH on nonformaldehyde durable press finishing of cotton fabric: FT-IR spectroscopy study: Part II: Formation of the anhydride intermediate. Text. Res. J..

[46] Yang Y., Li S. (1993). Silk fabric non-formaldehyde crease-resistant finishing using citric acid. J. Text. Inst..

[47] Mohsin M., Ramzan N., Ahmad S.W., Afzal A., Qutab H.G., Mehmood A. (2015). Development of Environment Friendly Bio Cross-Linker Finishing of Silk Fabric. J. Nat. Fibers.

[48] Khan M.M.R., Tsukada M., Gotoh Y., Morikawa H., Freddi G., Shiozaki H. (2010). Physical properties and dyeability of silk fibers degummed with citric acid. Bioresour. Technol..

[49] Chen Z.J., White M., Qiu Z.C. (2017). Investigation of Vacuum and Steam Treatments to Heat Treat and Sanitize Firewood-Grade Ash Logs and Ash Firewood. For. Prod. J..

[50] Chu Q.L., Song K., Bu Q., Hu J.G., Li F.Q., Wang J., Chen X.Y., Shi A.P. (2018). Two-stage pretreatment with alkaline sulphonation and steam treatment of Eucalyptus woody biomass to enhance its enzymatic digestibility for bioethanol production. Energy Conv. Manag..

[51] Lawther J.M., Sun R.C., Banks W.B. (1996). Effect of steam treatment on the chemical composition of wheat straw. Holzforschung.

[52] Fang L., Sun F.Y., Liu Q.B., Chen W.C., Zhou H., Su C.Z., Fang K.J. (2021). A cleaner production process for high performance cotton fabrics. J. Clean. Prod..

[53] Rekaby M., Salem A.A., Nassar S.H. (2009). Eco-friendly printing of natural fabrics using natural dyes from alkanet and rhubarb. J. Text. Inst..

[54] Li R.M., Wang L.L., Hao B.R., Wu M.H., Wang W. (2019). New thickener based on s-triazine di-sulfanilic xanthan for reactive printing of silk fabric with double-sided patterns. Text. Res. J..

[55] Murate H., Terasaki F., Shigematsu M., Tanahashi M. (2008). Improvement in the stretching property of paper yarn by shape memorization produced with high-pressure steam treatment. Sen-I Gakkaishi.

[56] Cai Z.S., Jiang G.C., Yang S.J. (2001). Chemical finishing of silk fabric. Color. Technol..

[57] Wang R., Zhu Y.F., Shi Z., Jiang W.B., Liu X.D., Ni Q.Q. (2018). Degumming of raw silk via steam treatment. J. Clean. Prod..

[58] Wang X.Q., Kluge J.A., Leisk G.G., Kaplan D.L. (2008). Sonication-induced gelation of silk fibroin for cell encapsulation. Biomaterials.

[59] Stathopulos P.B., Scholz G.A., Hwang Y.M., Rumfeldt J.A., Lepock J.R., Meiering E.M. (2004). Sonication of proteins causes formation of aggregates that resemble amyloid. Protein Sci..

[60] Grinstaff M.W., Suslick K.S. (1991). Air-filled proteinaceous microbubbles: Synthesis of an echo-contrast agent. Proc. Natl. Acad. Sci. USA.

[61] Gonzalez V., Wood R., Lee J., Taylor S., Bussemaker M.J. (2019). Ultrasound-enhanced hair dye application for natural dyeing formulations. Ultrason. Sonochem..

[62] McNeil S., McCall R. (2011). Ultrasound for wool dyeing and finishing. Ultrason. Sonochem..

[63] Velmurugan P., Shim J., Seo S.K., Oh B.T. (2016). Extraction of natural dye from coreopsis tinctoria flower petals for leather dyeing—An eco-friendly approach. Fibers Polym..

[64] Peila R., Grande G.A., Giansetti M., Rehman S., Sicardi S., Rovero G. (2015). Washing off intensification of cotton and wool fabrics by ultrasounds. Ultrason. Sonochem..

[65] Bahtiyari M.I., Duran K. (2013). A study on the usability of ultrasound in scouring of raw wool. J. Clean. Prod..

[66] Kadam V.V., Goud V., Shakyawar D. (2013). Ultrasound scouring of wool and its effects on fiber quality. Indian J. Fibre Text. Res..

[67] Mahmoodi N.M., Arami M., Mazaheri F., Rahimi S. (2010). Degradation of sericin (degumming) of Persian silk by ultrasound and enzymes as a cleaner and environmentally friendly process. J. Clean. Prod..

[68] Wang W.C., Pan Y., Gong K., Zhou Q., Zhang T.H., Li Q. (2019). A comparative study of ultrasonic degumming of silk sericin using citric acid, sodium carbonate and papain. Color. Technol..

[69] Devaraju S., Selvakumar N. (2012). Effect of Ozone Treatment on the Dyeing Properties of Mulberry and Tassar Silk Fabrics. J. Eng. Fibers Fabr..

[70] Sargunamani D., Selvakumar N. (2011). Comparative analysis of the effect of ozone treatment on the properties of mulberry and tassar silk fabrics. J. Text. Inst..

[71] Sargunamani D., Selvakumar N. (2007). Effects of ozone treatment on the properties of raw and degummed tassar silk fabrics. J. Appl. Polym. Sci..

[72] Sargunamani D., Selvakumar N. (2006). A study on the effects of ozone treatment on the properties of raw and degummed mulberry silk fabrics. Polym. Degrad. Stabil..

[73] Mahmoodi N.M., Moghimi F., Arami M., Mazaheri F. (2010). Silk Degumming Using Microwave Irradiation as an Environmentally Friendly Surface Modification Method. Fibers Polym..

[74] Wang F., Zhang Y.Q. (2017). Effects of alkyl polyglycoside (APG) on Bombyx mori silk degumming and the mechanical properties of silk fibroin fibre. Mater. Sci. Eng. C Mater..

[75] Bucciarelli A., Greco G., Corridori I., Pugno N.M., Motta A. (2021). A Design of Experiment Rational Optimization of the Degumming Process and Its Impact on the Silk Fibroin Properties. ACS Biomater. Sci. Eng..

[76] Yang Y., Zhang M., Tian W., Zhu C. (2017). Optimization of degumming process of and performance analysis of natural cassava silk. Adv. Text. Technol..

[77] Anis P., Capar G., Toprak T., Yener E. (2016). Sericin removal from silk fibers with eco-friendly alternative methods. Tekst. Konfeksiyon.

[78] Xiang W., Quan Q.Y., Ding J., Li K.C. (2011). Study on Mulberry Silk Degumming Process with Cold-Pad-Batch Using Tea Sapogenin, International Conference on Chemical Engineering and Advanced Materials.

[79] Wang Q., Ling S.J., Yao Q.Z., Li Q.Y., Hu D.B., Dai Q., Weitz D.A., Kaplan D.L., Buehler M.J., Zhang Y.Y. (2020). Observations of 3 nm Silk Nanofibrils Exfoliated from Natural Silkworm Silk Fibers. ACS Mater. Lett..

[80] Feng H.F., Wu Y.F., Feng X.M., Zhong L., Zhang F.X., Zhang G.X. (2018). A new acrylamide-glyoxal-based, formaldehyde-free elastic and stiffness finishing process for silk fabric. Text. Res. J..

[81] Parameswaran S. (2011). Silk—Queen of textiles. Colourage.

[82] Wang L., Lin J., Yuan J. (2005). Degumming process with environmental protection effect for two kinds of special silk fabrics. J. Text. Res..

[83] Zhu K.R., Kanu P.J., Claver I.P., Zhu K.X., Qian H.F., Zhou H.M. (2009). A method for evaluating Hunter whiteness of mixed powders. Adv. Powder Technol..

[84] (2008). Textiles-Test method Capillary rise. China Textile Criteria of FZ/T01071-2008.

[85] Elahi M.F., Guan G., Wang L., King M.W. (2014). Improved hemocompatibility of silk fibroin fabric using layer-by-layer polyelectrolyte deposition and heparin immobilization. J. Appl. Polym. Sci..

[86] Teuschl A.H., van Griensven M., Redl H. (2014). Sericin Removal from Raw Bombyx mori Silk Scaffolds of High Hierarchical Order. Tissue Eng. Part C Methods.

[87] Wang X., Qiu Y.W., Carr A.J., Triffitt J.T., Sabokbar A., Xia Z.D. (2011). Improved human tenocyte proliferation and differentiation in vitro by optimized silk degumming. Biomed. Mater..

[88] Carissimi G., Lozano-Perez A.A., Montalban M.G., Aznar-Cervantes S.D., Cenis J.L., Villora G. (2019). Revealing the Influence of the Degumming Process in the Properties of Silk Fibroin Nanoparticles. Polymers.

[89] Sahoo J.K., Choi J., Hasturk O., Laubach I., Descoteaux M.L., Mosurkal S., Wang B.Y., Zhang N.N., Kaplan D.L. (2020). Silk degumming time controls horseradish peroxidase-catalyzed hydrogel properties. Biomater. Sci..

[90] Kim H.J., Kim M.K., Lee K.H., Nho S.K., Han M.S., Um I.C. (2017). Effect of degumming methods on structural characteristics and properties of regenerated silk. Int. J. Biol. Macromol..

